# Prevalence of *Cryptosporidium parvum, Giardia intestinali*s and molecular characterization of group A rotavirus associated with diarrhea in children below five years old in Gaborone, Botswana

**DOI:** 10.11604/pamj.2020.37.159.25392

**Published:** 2020-10-14

**Authors:** Lineage Kurenzvi, Teresa Kibirige Sebunya, Tidimalo Coetzee, Giacomo Maria Paganotti, Mathias Vondee Teye

**Affiliations:** 1 Department of Biological Sciences, Faculty of Science, University of Botswana, Gaborone, Botswana,; 2 Botswana-University of Pennsylvania Partnership, Gaborone, Botswana,; 3 Division of Infectious Diseases, Perelman School of Medicine, University of Pennsylvania, Philadelphia, USA,; 4 Department of Biomedical Sciences, Faculty of Medicine, University of Botswana, Gaborone, Botswana

**Keywords:** Diarrhea, cryptosporidium, giardia, group A rotavirus, prevalence, genotype

## Abstract

**Introduction:**

Cryptosporidium, Giardia and rotaviruses are amongst the leading causes of acute gastroenteritis in children ≤5 years worldwide. The purpose of this study was to determine the occurrence of Cryptosporidium parvum, Giardia intestinalis and molecular characteristics of rotaviruses after Rotarix® introduction in Botswana.

**Methods:**

in this case study, 200 diarrheic stool specimens and 100 control samples from children under five years old were collected between March and November, 2017. Samples were analyzed by modified Ziehl Neelsen staining technique for cryptosporidium, wet mount procedure for Giardia and negative samples were confirmed by immunochromatographic assay. Specimens were analyzed for rotavirus by ELISA, PAGE, RT-PCR, sequencing of VP7 and VP4 antigen followed by phylogenetic analysis.

**Results:**

prevalence rates of 20.5%, 16.5% and 11.0% in diarrhea cases were observed for Cryptosporidium parvum, Giardia intestinalis and rotavirus, respectively. Four percent of diarrheic specimens had multiple infections. The predominant rotavirus genotype was GIP[8] (7/15) followed by G2P[4] (2/15) and G3P[8] (1/15). Twenty percent of specimens were non-typeable. One mixed strain, G1+G2P[4,8] (2/15), was detected. Phylogenetic analysis of VP4 and VP7 sequences clustered Botswana rotavirus strains within G1 lineages 1 and 2, G3 lineage 1, P[8] lineage 3 and P[4] lineage 5 together with Southern African strains.

**Conclusion:**

this study provides important information on occurrence and demographic risk groups for Cryptosporidium parvum, Giardia intestinalis and rotavirus in young children as well as genetic diversity of rotaviruses after vaccine introduction in Botswana. Constant monitoring of circulating rotavirus strains is essential in assessing effectiveness of current vaccines in Botswana.

## Introduction

Diarrhea is a common disease in developing countries especially in regions where there is poor sanitation and hygiene [1]. In developed countries, diarrhea is rarely fatal except in people at extremes of age and immunocompromised individuals. Globally, most under-fives suffer 3 to 4 episodes of diarrhea in a year [2]. Prolonged and recurrent diarrhea leads to malnutrition and poor growth in children below the age of five years [3]. The introduction of water and sanitation hygiene (WASH) programs, Oral Rehydration Therapy (ORT), vaccinations and adequate nutrition measures gradually reduced mortality and morbidity due to diarrhea in children [4]. However, there are rising concerns about potential increase of diarrheal deaths in Botswana and other nations due to increasing temperatures that pose negative effects on surface water resource [5,6].

*Cryptosporidium* and *Giardia* are the most common protozoan pathogens that cause gastroenteritis worldwide. Both parasites are considered emerging opportunistic pathogens responsible for diarrheal morbidity globally [7]. By 2016, *Cryptosporidium* was the fifth leading cause of diarrheal mortality, responsible for approximately 44.8 million episodes of diarrhea and 48,300 annual deaths in children below the age of five [8]. Prevalence rates of *Cryptosporidium* ranges from 2 to 60% in Botswana, affecting children ≤24 months of age more than any other age groups [9,10]. This highlights the need to revisit and evaluate the burden of cryptosporidiosis in Botswana.

In 2010, the World Health Organization estimated that *Giardia* was responsible for 28.2 million cases of diarrhea globally [11]. *Giardia* outbreaks were also reported in 37% of global waterborne transmission of protozoan parasites between 2011 and 2016 [12]. The occurrence of *Giardia* ranges from <1% to 72% in Africa [13] and 1 to 10% in Botswana [14,15]. Although prevalence rates of *Giardia* infections are lower in Botswana, some studies reveal that chronic giardiasis modulates symptoms of rotavirus infection and co-infection in giardiasis patients intensifies severity of diarrhea [16]. Association between *Giardia* and rotavirus infections has not been well investigated in Botswana, yet higher case fatality ratios have been previously reported in diarrheal children [17].

The Global Burden of Diseases (GBD) reported rotavirus as the leading cause of all diarrhea deaths in 2016, leading to approximately 128,515 deaths in children under five years old [18]. In 2009, two live attenuated oral rotavirus vaccines, RotaTeq™ and Rotarix®, were recommended by World Health Organization in national immunization programmes [19]. The introduction of Rotarix®, increased protection against rotavirus associated illnesses among children in Botswana, but changing diversity of circulating strains had been evident in most studies conducted in the post vaccination era [10,20]. Genetic reassortments of rotavirus strains are possible under vaccine pressure [21] and continuous rotavirus surveillance studies are important in evaluating effectiveness of current vaccines on rotavirus genotypes circulating in Botswana.

In this study, we aimed at determining the prevalence of *Giardia intestinalis, Cryptosporidium parvum* and rotavirus in children below the age of five years in Gaborone, Botswana. This study was also established to investigate the distribution pattern of group A rotavirus genotypes circulating in young children up to five years of age who were presented with diarrhea in Gaborone.

## Methods

**Study design, study site and study population:** this case study was conducted from March to November 2017 in Gaborone, the largest and capital city of Botswana. Diarrheal participants were children below the age of five years whose stool samples were collected at Princess Marina Hospital, Bokamoso Private Hospital and Diagnofirm medical microbiological laboratories. Non-diarrheic participants were children below the age of five from the pediatric ward of Princess Marina Hospital and Gaborone Child Welfare clinics.

**Ethical clearance:** this study was approved by the Botswana Ministry of Health in Gaborone (ref. no: HPDME 13/18/1), the Institutional Review Board of University of Botswana (ref. no: UBR/RES/IRB/GRAD/297) and Princess Marina Hospital Research and Ethics Committee (ref. no: PMH5/79 [302-1-2017]). Permission was also obtained from all the authorities of wards and clinics. Parental consent was requested from parents/guardians of participants where applicable.

**Specimen collection:** two hundred stool samples of children presented with diarrhea were collected from Princess Marina Hospital, Bokamoso Private Hospital and Diagnofirm medical microbiological laboratories. For controls, 100 stool specimens from non-diarrheic children were collected from parents/guardians of children from Princess Marina Hospital pediatric ward and Gaborone Child Welfare clinics. Samples were collected in sterile containers, placed in an ice box before transportation to the virology laboratory of University of Botswana. Fecal specimens were divided into two aliquots before storage. Specimens intended to be analyzed for parasites were examined microscopically and stored at -20°C without any preservative. A 10% suspension was made on stool specimens intended to be analyzed for rotavirus. Suspensions were stored at 4°C until the time of analysis.

**Detection of *Cryptosporidium* and *Giardia* cysts:** *Giardia* cysts were detected microscopically by the wet mount procedure [22]. The modified Ziehl Neelsen technique was used to stain *Cryptosporidium* oocysts in fecal specimens [23]. All smears were viewed under the light microscope at 10x objective followed by 40x objective. Confirmation of absence of *Cryptosporidium parvum* and *Giardia intestinalis* in stool samples that tested negative was done by using a one-step Crypto+Giardia combo card (CerTest Biotec S.L., Zaragoza, Spain).

### Laboratory investigations for rotavirus

**Polyacrylamide gel electrophoresis:** ELISA kits for human rotavirus group A (IVD Research Inc., Carlsbad, CA, USA) were used to detect the presence of rotavirus antigen, following manufacturer´s instructions. Viral RNA was extracted from 16 strongly ELISA positive samples using the phenol/chloroform method. Migration patterns of the rotavirus segmented genome was then detected by polyacrylamide gel electrophoresis (PAGE) in 10% acrylamide slab gels with a 4% stacking gel. A discontinuous buffer system without sodium dodecyl-sulphate was used [24].

**Reverse transcriptase-polymerase chain reaction:** viral RNA intended to be used for reverse transcriptase-polymerase chain reaction (RT-PCR) was extracted from fecal suspensions using the ZR viral RNA kit (Zymo Research, Orange, CA, USA) following manufacturer´s procedures. The protoscript II cDNA synthesis kit (New England Biolabs, USA) was used for cDNA synthesis according to the manufacturer´s instructions. The cDNA generated from reverse transcription was used as a template for PCR. Specific primer pairs con2/con3 were used for consensus PCR for VP4. Reactions were carried out in a thermal cycler with the following conditions: 2 minutes at 94°C; 35 cycles of 1 minute at 94°C, 1 minute at 50°C, 1 minute at 72°C; a final extended step of 7 minutes at 72°C, then held at 15°C. VP4 genotyping was done by using the forward primer con3 and a cocktail of primers specific to human rotavirus P-genotype P[4], P[6], P[8], P[9] and P[10]. The solution was denatured at 94°C for 4 minutes followed by 30 cycles of PCR at 94°C for 1 minute, 45°C for 2 minutes and 72°C for 1 minute. Reactions were extended for 7 minutes at 72°C, then held at 15°C.

Beg9/End9 primer pair was used for the outer PCR for VP7. Reactions were cycled under the following conditions: 2 minutes at 94°C; 35 cycles of 1 minute at 94°C, 1 minute at 52°C, 1 minute at 72°C; with a final extension of 7 minutes at 72°C, then held at 15°C. Multiplex PCR for VP7 was done using the reverse primer End9 with a cocktail of primers specific to G1, G2, G3, G4, G8 and G9. PCR reactions were performed in a thermal cycler with the following conditions: 2 minutes at 94°C; then 35 cycles of 1 minute at 94°C, 1 minute at 50°C, 1 minute at 72°C; with a final extended step of 7 minutes at 72°C, held at 15°C. All VP4 and VP7 amplicons were visualized by electrophoresis (alongside a 100bp DNA ladder) on a 1.5% agarose gel stained with ethidium bromide then photographed under ultraviolet light.

**Nucleotide sequencing of RT-PCR amplicons and phylogenetic analysis:** amplicons from 10 specimens were selected for sequence analysis. Sequencing was done using the same primers for RT-PCR. PCR products were purified by the Exonuclease and Shrimp Alkaline Phosphatase (ExoSAP) procedure according to manufacturer´s protocol. Fragments were sequenced using the nimagen, BrilliantDye™ terminator cycle sequencing kit V3.1, according to manufacturer´s instructions. Labelled products were then cleaned with the ZR-96 DNA sequencing clean-up kit (Zymo Research, Orange, CA, USA) and later injected on the Applied Biosystems ABI 3500XL genetic analyser with a 50cm array, using POP7.

### Data analysis

**Statistical analysis:** to summarize data generated from serological tests and electrophoresis, descriptive statistics (frequencies and percentages) were calculated for all categorical variables using Microsoft Excel Software, Version 2013 (Microsoft Corporation, USA). The Chi-square test was used to compare categorical variable proportions and statistical significance. A P-value of less than 0.05 was considered to be statistically significant.

**Phylogenetic analysis:** after sequencing, the chromatograms of sequences were assembled and visually analyzed using BioEdit software version 7.2.5 [25]. Identification of genotypes of the sequences was achieved by comparison with reference sequences available in the NCBI GenBank database using BLAST [26]. Multiple sequence alignments was carried out using Clustal W [27] incorporated in BioEdit software version 7.2.5 [25]. Phylogenetic analysis was done in MEGA software version 6.06 [28] using a distance-based neighbor-joining of the Kimura 2-parameter nucleotide substitution model [29]. The robustness of each tree branch was tested by performing 1,000 bootstrap replicates.

Nucleotide sequence accession numbers: the nucleotide sequences of group A rotaviruses of this study were deposited in GenBank and assigned accession numbers MT364283 to MT364302 (Table 1).

**Table 1:** VP7 and VP4 sequences obtained in this study and their GenBank accession numbers

Isolate	G genotype (acc. no.)	P genotype (acc. no.)
RVA/human-wt/BWA/014/2017	G1 (MT364283)	P[8] (MT364293)
RVA/human-wt/BWA/048a/2017	G1 (MT364284)	P[8] (MT364294)
RVA/human-wt/BWA/048b/2017	N/A	P[4] (MT364295)
RVA/human-wt/BWA/063/2017	G1 (MT364285)	P[8] (MT364296)
RVA/human-wt/BWA/075a/2017	G1 (MT364286)	P[8] (MT364297)
RVA/human-wt/BWA/075b/2017	N/A	P[4] (MT364298)
RVA/human-wt/BWA/141/2017	G1 (MT364287)	P[8] (MT364299)
RVA/human-wt/BWA/145/2017	G1 (MT364288)	P[8] (MT364300)
RVA/human-wt/BWA/149/2017	G3 (MT364289)	P[8] (MT364301)
RVA/human-wt/BWA/161/2017	G1 (MT364290)	P[8] (MT364302)
RVA/human-wt/BWA/163/2017	G1 (MT364291)	P[8] (MT364303)
RVA/human-wt/BWA/165/2017	G1 (MT364292)	P[8] (MT364304)

Acc. no. =GenBank accession number; N/A = not applicable (sequencing was not successful)

## Results

**Figure 1:**
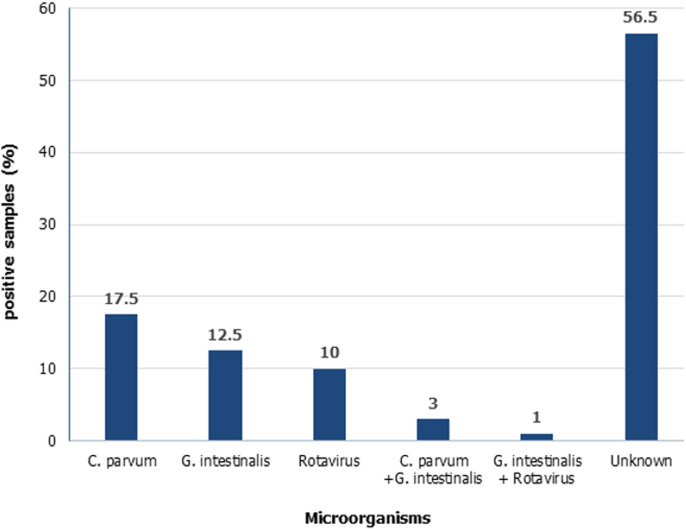
prevalence of *Cryptosporidium parvum, Giardia intestinalis* and rotaviruses in children under five years old in Gaborone

**Epidemiology of *Cryptosporidium parvum, Giardia intestinalis* and rotavirus:** the most common pathogens in this study were parasites (Figure 1). *Cryptosporidium parvum* was detected in 20.5% (41/200) of all the diarrheic samples, and 61% (25/41) of positive samples came from male children. Most *Cryptosporidium parvum* cases were from children ≤36 months of age (32/41, 78%, P=0.100). The peak month of cryptosporidium infections was October and cases dropped during the winter season. *Giardia intestinalis* was found in 16.5% (33/200) of children suffering from diarrhea. No significant association was found between prevalence and gender, although it affected more females than males (18/33, 54.5%, P=0.895). More cases of *Giardia intestinalis* were from children aged ≤36 months (27/33, 81.8%, P=0.449). Slight fluctuations in occurrence of *Giardia intestinalis* were seen frequently throughout the entire study period.

Rotavirus was the least detected pathogen (11.0%) in all 200 samples of children with diarrhea. Fifteen (15) samples (68.2%, P=0.93) were from children aged ≤24 months old. Rotavirus affected male (11/22, 50%) and females (11/22, 50%) equally. Infections were at peak in April and in July. Three percent (3%) of diarrheic samples yielded both *Giardia intestinalis* and *Cryptosporidium parvum*, while 1% had both *Giardia intestinalis* and rotavirus.

**Rotavirus electrophoretypes and genotypes:** most of the isolates revealed the 4-2-3-2 gene migration pattern of human group A rotavirus. Only two major patterns were identified from diarrheic samples, long (9/16, 56.25%) and short (4/16, 25%) electrophoretypes. Two samples (12.5%) had more than 11 gene segments and no clear migration was found in 6.25% (1/15) of diarrheic samples. Only 3 G genotypes, G1 (9/15, 60%), G2 (4/15, 26.7%) and G3 (1/15, 6.7%) were observed. The most predominant genotype was GIP[8] (6/15, 40%) followed by G2P[4] (2/15, 13.3%) and G3P[8] (1/15, 6.7%). Uncommon rotavirus genotype G1P[6] was detected in approximately 7% (1/15) by RT-PCR. Two cases (13.3%) were found to have an uncommon combination G1+G2P [4,8] and 20% of the specimens tested by PCR were untypeable.

**Phylogenetic analysis of VP4 and VP7 genes:** sequence alignment and phylogenetic analysis of the VP7 genes of the 10 rotavirus strains that were successfully sequenced showed 96-99% nucleotide sequence identity after being compared to corresponding G1 and G3 strains internationally. The VP4 gene sequences of rotavirus strains from this study showed nucleotide sequences with 96-97% identity with regional and international P[4] and P[8] strains. One sample that was thought to be G1P[6] by RT-PCR was confirmed to be G1P[8] after sequencing. Sequencing of the G2 strains detected by RT-PCR was not successful, therefore no sequence data was obtained for phylogenetic analysis.

**Figure 2:**
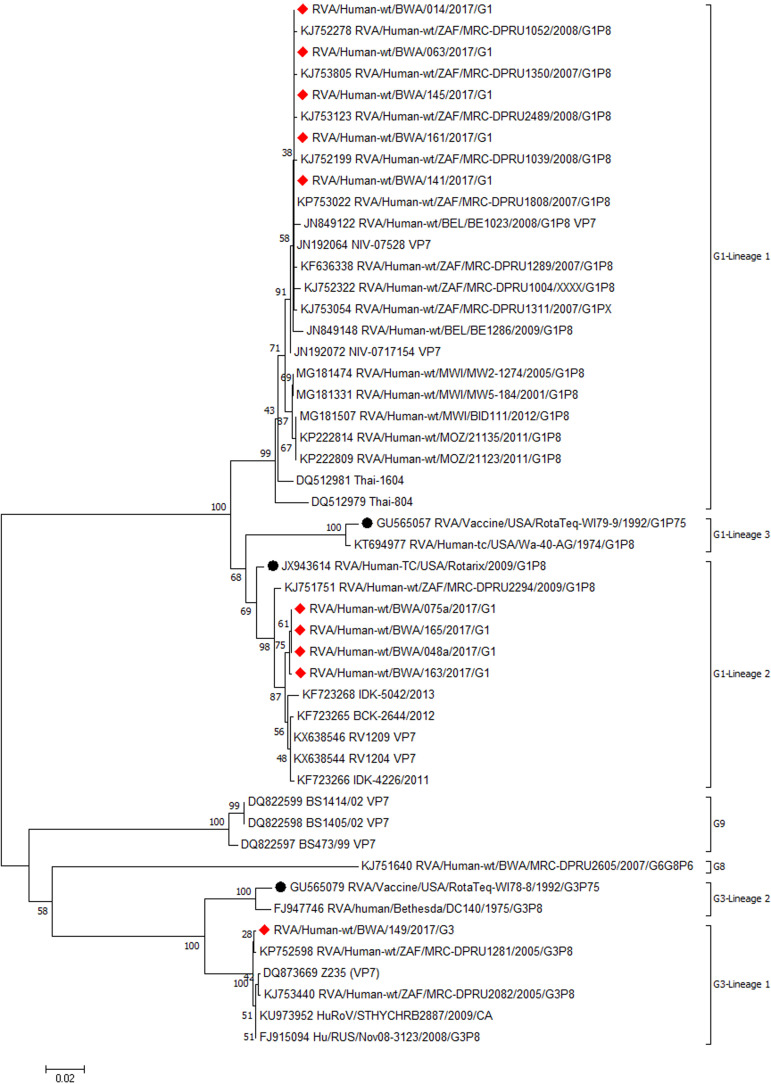
phylogenetic analysis of VP7 nucleotide sequences of rotaviruses in Botswana and international strains based on the neighbor-joining method; the 2017 strains from Botswana are indicated by red diamonds; vaccine strains are indicated by circles; bootstrap test (1000 replicates) values are shown on the branches

The G1 strains of Botswana clustered G1 sequences into 2 lineages (Figure 2). Five of the G1 strains (RVA/human-wt/BWA/O14/2017/G1, RVA/human-wt/063/2017/G1, RVA/human-wt/BWA/141/2017/ G1, RVA/human-wt/BWA/145/2017/G1 and RVA/human-wt/BWA/161/2017/G1) clustered in lineage 1 with strains from South Africa (KJ753805, KJ752278, KJ753123 and KP753022), Malawi (MG181474, MG181331 and MG181507), Mozambique (KP222814, KP222809), Thailand (DQ512974, DQ512981), India (JN192064) and Belgium (JN849122). The other four G1 sequences (Figure 2) from this current study: RVA/human-wt/BWA/048a/2107/G1, RVA/human-wt/BWA 075a/2017/G1, RVA/human-wt/BWA/163/2017/ G1 and RVA/human-wt/BWA/165/2017/G1 fell into lineage 2 and exhibited 97 to 99% identity among themselves and clustered with strains discovered earlier in India (KX638546), South Africa (KJ751751) and China (DQ873669). The G3 sequence of this study (RVA/human-wt/BWA/149/2017/G3) was assigned to G3 lineage 1 and was closely related to G3 strains previously identified in South Africa (KP752598, KJ753186 and KJ753440) and China (DQ873669) (Figure 2). A distant relationship was found between all VP7 strains with both Rotarix (G1 lineage 2) and RotaTeq (G3 lineage 2).

**Figure 3:**
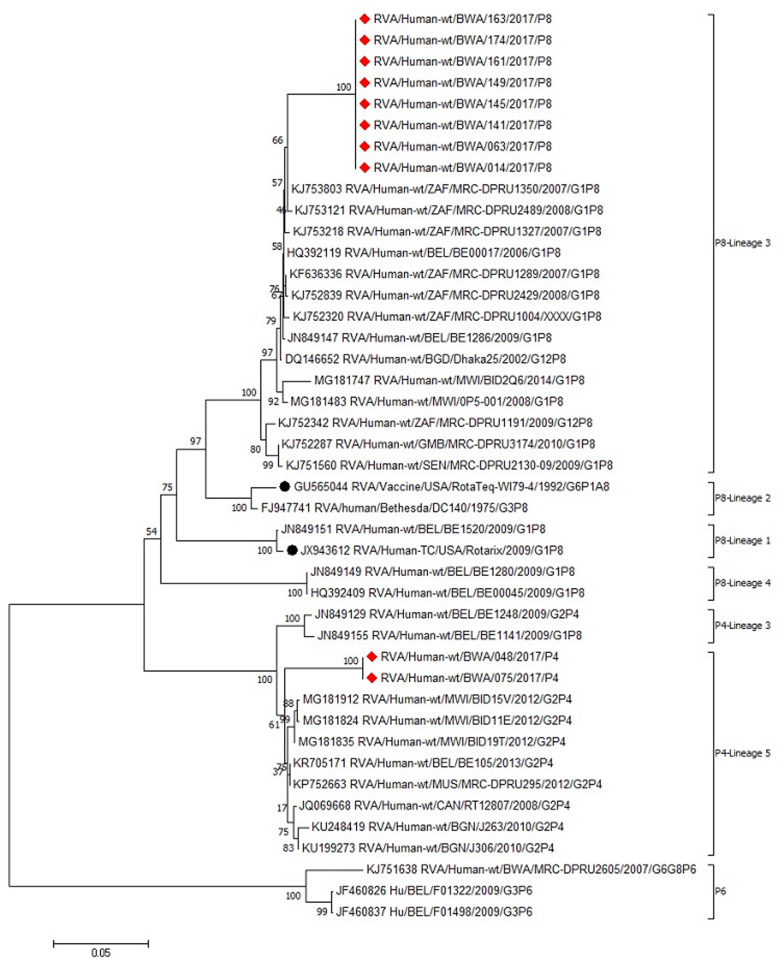
phylogenetic analysis of VP4 nucleotide sequences of rotaviruses in Botswana and international strains based on the neighbor-joining method; the 2017 strains from Botswana are indicated by red diamonds; vaccine strains are indicated by circles; bootstrap test (1000 replicates) values are shown on the branches

Botswana VP4 sequences (Figure 3) were assigned to P[8] genotype lineage 3 and P[4] lineage 5 and were all distantly related to both Rotarix (P[8] lineage 2) and RotaTeq (P[8] lineage 1). All the observed P[8] sequences in this study shared 100% nucleotide sequence identity with each other and clustered in P[8] lineage 3 with sequences from South Africa (KJ753803, KJ753121), Malawi (MG181483), Gambia (KJ752287), Senegal (KJ751560) and Belgium (HQ392119, JN849147). Isolated P[4] strains (RVA/human-wt/BWA/048b/2017/P4 and RVA/human-wt/BWA/075b/2017/P4) clustered with other sequences from Malawi (MG181912 and MG181835), Mauritius (KP752663) and Belgium (KR705171).

## Discussion

This present study determines the prevalence of *Cryptosporidium parvum, Giardia intestinalis* and rotavirus among children below the age of five years in Gaborone. Prevalence of 20.5% for *Cryptosporidium parvum* and 16.5% for *Giardia intestinalis* observed in this study were higher than those reported in similar studies previously conducted in Botswana [10,14,15]. A possible explanation of this discrepancy can be variations in research set ups, sample population, environmental condition of study locations, sample size as well as methods used for detection. Furthermore, climate change recently increased water scarcity in cities like Gaborone, promoting the use of contaminated household water storage and increase exposure to parasitic infections [30].

Most *Cryptosporidium parvum* and *Giardia intestinalis* infections occurred primarily in children ≤36 months of age, and this correlates with worldwide reports that cryptosporidiosis and giardiasis affects children in lower age groups more frequently than any other age group [8]. High prevalence of *Giardia intestinalis* and *Cryptosporidium parvum* in younger children may be as a result of increased exposure to contaminated municipal water and increased contact with contaminated surfaces. Lack of previous exposure to *Giardia intestinalis* also render young children to be more susceptible to infection [31].

Approximately 60% of male children were significantly affected by cryptosporidiosis. Most researchers associate cryptosporidiosis and giardiasis distribution to social, cultural and behavioral differences between male and female children [32,33]. However, no apparent association was established between gender and giardiasis in this study. Lack of association of prevalence of *Giardia intestinalis* with gender might have been caused by a smaller sample size used in this current study. Although giardiasis show fluctuations from spring to winter, the peak months of both cryptosporidiosis and giardiasis transmission coincided with the hot rainy season. This finding agrees with prior reports from Chobe district that suggested that cryptosporidiosis and giardiasis coincide with high precipitation periods [5].

In Botswana, rotavirus has been reported as the most prevalent pathogen of diarrhea in studies where both *Cryptosporidium* species and rotavirus had been investigated before rotavirus vaccine introduction [34]. Lower prevalence of rotavirus than *Cryptosporidium* observed in this study can be as a result of high efficiency of the monovalent rotavirus vaccine Rotarix® currently used in Botswana [20]. Similar trends were reported in other African countries that engaged Rotarix in their immunization programmes [35,36]. A higher proportion of rotavirus in children ≤24 months old observed in this current study is consistent with some previous studies in Botswana [20,34] and other nations like South Rajasthan and China [37,38]. Peak prevalence of rotavirus infections in colder months agree with previous findings in local studies [39-41] although some researchers deduced that there is no unifying explanation for the global varying seasonality of rotavirus [42].

Only 3 G genotypes (G1, G2 and G3) and 2 P genotypes (P[4] and P[8]) were observed in this study polyacrylamide gel electrophoresis confirmed that all the G1 were associated with long electrophoretypes whilst all G2 and G3 showed short electrophoretic patterns. These migration pattern correlate with findings from other researchers in Botswana [40,41], Eastern and Southern African countries [43]. The most predominant genotype of rotavirus in this study was G1P[8], detected in 40% of all sequenced samples. In Botswana G1P[8] was one of the major isolated strains before rotavirus vaccination [41]. G1P[8] gradually decreased in Botswana after Rotarix® introduction, but later reappeared the following year [44]. Global epidemiological studies on rotavirus also identified G1P[8] as one of the most prevalent strain even after vaccine introduction [45]. Phylogenetic analysis of G1 sequences assigned five of Botswana G1P[8] strains into lineage 1 and were closely related to some G1P[8] strains discovered in South Africa, Malawi and Mozambique. This observation indicates that these G1P[8] strains might be direct descendants of previous strains from neighboring countries rather than emergence from point mutations. The other four G1 sequences formed a separate cluster in lineage 2 together with G1P[8] strains previously observed in India. This observation indicates that these might have been introduced in Botswana by continental migration, genetic reassortment or mutation.

In this study, G2P[4] was the second leading cause of rotavirus associated diarrhea, although it was the leading cause of rotavirus acquired illnesses in a post vaccination surveillance study in Botswana [20]. Circulating rotavirus genotypes may vary on a yearly basis and their occurrence may also be affected by natural cyclic patterns as well as seasonal variations [46,47], highlighting the importance of surveillance studies of rotavirus diseases. Phylogenetic analysis of the P[4] sequences clustered the G2P[4] strains of the current study in P[4] lineage 2 with other strains from Malawi, Mauritius and Belgium, indicating the derivation of those strains from a common origin. However, phylogenetic analysis of G2 sequences of this study was impossible. This might have been caused by poor DNA quality and low viral titers. G3 strain for human rotavirus was first detected in Botswana in the early 2000s and in most cases was associated with P[6] and other untypeable P genotypes [40,41]. G3 cases drastically declined in the post vaccination era [20] and was the least detected in this present study. Similar observations were reported in studies conducted in Eastern and Southern African countries after Rotarix vaccine introduction [43]. This suggests that Rotarix® may have high efficacy against G3P[8] as well as cross-protection against serotypes not included in the vaccine´s composition. The single G3P[8] of this current study was closely related to G3P[8] strains circulating in South Africa and China indicating that they might have descended from a common ancestor.

In many African countries, rotavirus strains of G types G1, G2, G8, G9 and P types P[4], P[6], P[8], P[9] had been isolated from drinking water, which is one of the major environmental factors that promote mixed infections in developing countries [48,49]. Phylogenetic analysis of samples with mixed infections of this study revealed that G1+G2 P[4,8] was as a result of infection by both G1P[8] and G2P[4] strains closely related to previously identified strains from South Africa and Malawi, respectively. However, complete genome sequencing of the strains would be a necessity to determine the potential of any natural reassortment.

## Conclusion

*Cryptosporidium parvum, Giardia intestinalis* and rotavirus are most important pathogens of diarrheal diseases in young children. Most *Cryptosporidium parvum* and *Giardia intestinalis* transmissions coincided with the hot, rainy season of the year while rotavirus infections were at peak in winter. Further research is required to understand seasonal peaks of *Cryptosporidium parvum, Giardia intestinalis* and rotavirus to establish more intervention measures that disrupt transmission in young children. Rotavirus strains circulating in Botswana are similar to those frequently occurring worldwide. Phylogenetic analyses of the isolated rotavirus strains of Botswana indicate that they are distantly related to Rotarix, but there might be transmission between Botswana and some southern African countries, especially South Africa and Malawi. To obtain more understanding of the epidemiology of rotavirus strains in the region and to assess the effectiveness of vaccines strains currently being used in the country, more and continuous surveillance studies of circulating rotavirus genomes need to be done. Whole genome sequencing of circulating rotavirus strains would be essential to determine the extent of genetic variation and the relatedness of Botswana rotavirus strains with those described worldwide.

### What is known about this topic

- Cryptosporidium, Giardia and rotaviruses mostly affects children younger than 2 years in some parts of Botswana;
- The distribution of rotavirus genotypes circulating in young children changed after vaccine implementation in Botswana.

### What this study adds

- This study confirmed that Cryptosporidium, Giardia and rotavirus are more prevalent in the early stages of life than the later;
- This study provides information on the sequences of circulating rotavirus strains in young children in Gaborone in 2017;
- This present study confirmed that most rotavirus strains circulating in children below 5 years old in Gaborone are related to strains previously identified in other Southern African countries.
